# Ulipristal Acetate Interferes With Actin Remodeling Induced by 17β-Estradiol and Progesterone in Human Endometrial Stromal Cells

**DOI:** 10.3389/fendo.2018.00350

**Published:** 2018-06-27

**Authors:** Jorge E. Shortrede, Maria M. Montt-Guevara, Gisela Pennacchio, Michele Finiguerra, Andrea Giannini, Alessandro D. Genazzani, Tommaso Simoncini

**Affiliations:** ^1^Molecular and Cellular Gynecological Endocrinology Laboratory, Department of Clinical and Experimental Medicine, University of Pisa, Pisa, Italy; ^2^Institute of Experimental Medicine and Biology of Cuyo, Technology and Scientific Center (CCT)-National Research Council of Argentina, Mendoza, Argentina; ^3^Department of Obstetrics and Gynecology, Center for Gynecological Endocrinology, University of Modena and Reggio Emilia, Modena, Italy

**Keywords:** Ulipristal acetate (UPA), endometrial stromal cells (ESC), progesterone (P4), 17β-estradiol (E2), actin remodeling

## Abstract

Ulipristal acetate (UPA) is a selective progesterone receptor modulator (SPRM) used for emergency contraception and for the medical management of symptomatic uterine fibroids (UF). Treatment with UPA turns in amenorrhea and UF volume reduction. Treatment with UPA is associated with the frequent development of benign, transitory endometrial changes known as SPRM-associated endometrial changes (PAECs). Why PAECs develop and their biological or cellular basis is unknown. Sex steroids, including estrogen and progesterone, are established modulators of the actin cytoskeleton in various cells, including endometrial cells. This explains several morphological and functional changes in endometrial cells. We thus hypothesized that UPA may alter the appearance of the endometrium by interfering with the actions of 17β-estradiol (E2) or progesterone (P4) on actin dynamics. We isolated and cultured human endometrial stromal cells (ESC) from endometrial biopsies from healthy fertile women. Treatment with E2 or P4 stimulated visible actin rearrangements with actin remodeling toward the membrane. Activation through phosphorylation of the actin regulatory proteins, Moesin, and focal adhesion kinase (FAK), hacked actin remodeling induced by E2 and P4. Membrane re-localization of Paxillin and Vinculin were also induced by E2 and P4, showing the formation of focal adhesion complexes. All these E2 and P4 actions were inhibited by co-treatment with UPA, which was otherwise inactive if given alone. The cytoskeletal changes induced by E2 and P4 turned into increased motility of ESC, and UPA again blocked the actions E2 and P4. In conclusion, we find that UPA interferes with the cytoskeletal actions of E2 and P4 in ESC. This finding helps understanding the mode of actions of SPRMs in the endometrium and may be relevant for other potential clinical applications of UPA.

## Introduction

Progesterone receptor (PR) modulators are under clinical development to treat conditions such as breast cancer, endometriosis, dysfunctional uterine bleeding, and uterine fibroids (UF) (1, 2). Since 2012, Ulipristal acetate (UPA) has been approved in Europe for treatment of UF (3, 4). Ulipristal acetate is a 19-norprogesterone derivative that inhibits proliferation and stimulates apoptosis of leiomyoma cells without affecting normal myometrial cells (3). Its administration is associated with development of a rapid and stable amenorrhea, which can be in part explained by interference with the hypothalamus-pituitary-ovarian axis. Currently, UPA has two major indications in several countries: emergency contraception (4, 5) and treatment of symptomatic fibroids (6–8).

Prospective, placebo-controlled, Phase III clinical trials using UPA in the management of UF (PEARL I, II, III, and IV) have proved the efficacy and safety of UPA (9). It has been observed that the use of UPA is associated with endometrial modifications, known as selective progesterone receptor modulator (SPRM)—associated endometrial changes (PAEC) (10, 11). PAECs are benign and reversible with the interruption of the therapy (9). Nevertheless, the significance of those changes nor the biological basis are unknown.

Endometrial remodeling is the term used to indicate the set of cellular processes that dynamically happen during the menstrual cycle, pregnancy, or in the development of endometrial disorders (12, 13). These biological phenomena are identified with dynamic changes in cell architecture and the progressive modifications of the cell-cell and cell-matrix interactions that determine the macroscopic endometrial changes (14). One of the processes related with such phenomena is the ability of cells to remodel their actin cytoskeleton. Modifications of actin fibers architecture drives cell membrane reshaping and movement (15, 16). The major proteins involved in the regulation of focal adhesion complex formation and actin polymerization/stabilization are Focal Adhesion kinase (FAK), Paxillin (a scaffolding protein associated to focal adhesions), and Membrane-Organizing Extension Spike protein (Moesin).

The aim of the present study was to establish whether UPA affects the morphology of human ESC with special attention on how UPA acts on actin cytoskeleton rearrangement, FAK/Paxillin/Moesin activation and cell migration. To this extent, we studied the effects of UPA alone or, in the presence of progesterone or 17β-estradiol.

## Materials and methods

### Endometrial cell sampling

Endometrial samples were obtained from eight women undergoing diagnostic operative hysteroscopy procedures. All samples were collected during the follicular phase, based on the last menstrual period and histological examination of the samples, between December 2015 and June 2016. All women were under 45 years with regular menstrual cycles for the past 12 months. Women who underwent endocrine treatments (oral contraceptives, progestins, GnRH analogues or antagonists) in the last 3 months or with endometriosis or endometrial cancer were excluded from the study.

This study was carried out in accordance with the recommendations of the Good Clinical Practice (ICH/GCP), Ministerial Decree of 1997. The protocol was approved by the Regional Ethics Committee for Clinical Trials, North West Wide Area. All subjects gave written informed consent in accordance with the Declaration of Helsinki.

### Cell preparation, culture, and characterization of human endometrial cells

#### Cell preparation and culture

After tissue collection, samples were rapidly immersed in phenol-red free Dulbecco's minimal essential medium (DMEM, Gibco, USA) with 10% FBS (Gibco, USA) plus antibiotic-antimycotic (ATB, Gibco, USA), and immediately processed for endometrial cell isolation (17, 18). Under a laminar flow sterile hood, biopsy samples were mechanically chopped into 1–2 mm^3^ and washed with fresh medium to remove blood and mucosa. Then, the slices were incubated with 10 ml of pre-warmed 0.2% type II collagenase (Sigma-Aldrich, USA) for 60 min at 37°C in a shaking rotor machine. Following digestion, cell clumps were mechanically dispersed by aspiration through a Pasteur pipette. After 10 min of differential sedimentation at single gravity, ESC were separated from large clumps of epithelium. The top 8 ml of medium, containing predominantly stromal cells, were collected and centrifuged (200 g for 5 min). The stromal-enriched fraction was washed three times with fresh medium. Lastly, ESC were allowed to adhere selectively to 25 cm^2^ flask for 15 min in phenol-red free DMEM-F12 (Gibco, USA) supplemented with 10% FBS and ATB. Afterward, non-attached endometrial epithelial cells (EEC) were removed and a purified stromal preparation was obtained. Endometrial stromal cells (ESC) were cultured to sub-confluence in DMEM-F12 with 10% FBS and ATB and were used between passages 3 and 10.

#### Cell treatments

Before each experiment, medium was replaced for 48 h with medium containing charcoal stripped-FBS (CS-FBS, which is steroid-deprived FBS, Lonza, Switzerland), or medium without FBS, when experiments were conducted to study non-transcriptional effects. When an inhibitor was used, the active treatments were added 1 h later. Control cells always received the same amount of ethanol (solvent for UPA/P4/E2 0.01% final concentration).

Ulipristal acetate (10^−9^ to 10^−7^ M) was kindly provided by Gedeon Richter, UK. Progesterone (P4, 10^−9^ to 10^−7^ M), 17β-estradiol (E2, 10^−9^ to 10^−7^ M), the PR Antagonist Mifepristone (MIF, 10^−5^ M), and the Estrogen Receptor (ER) Antagonist Fulvestrant (ICI 182,780, 10^−6^ M) were purchased from Sigma-Aldrich, USA.

#### Cell characterization

**Figure 1:**
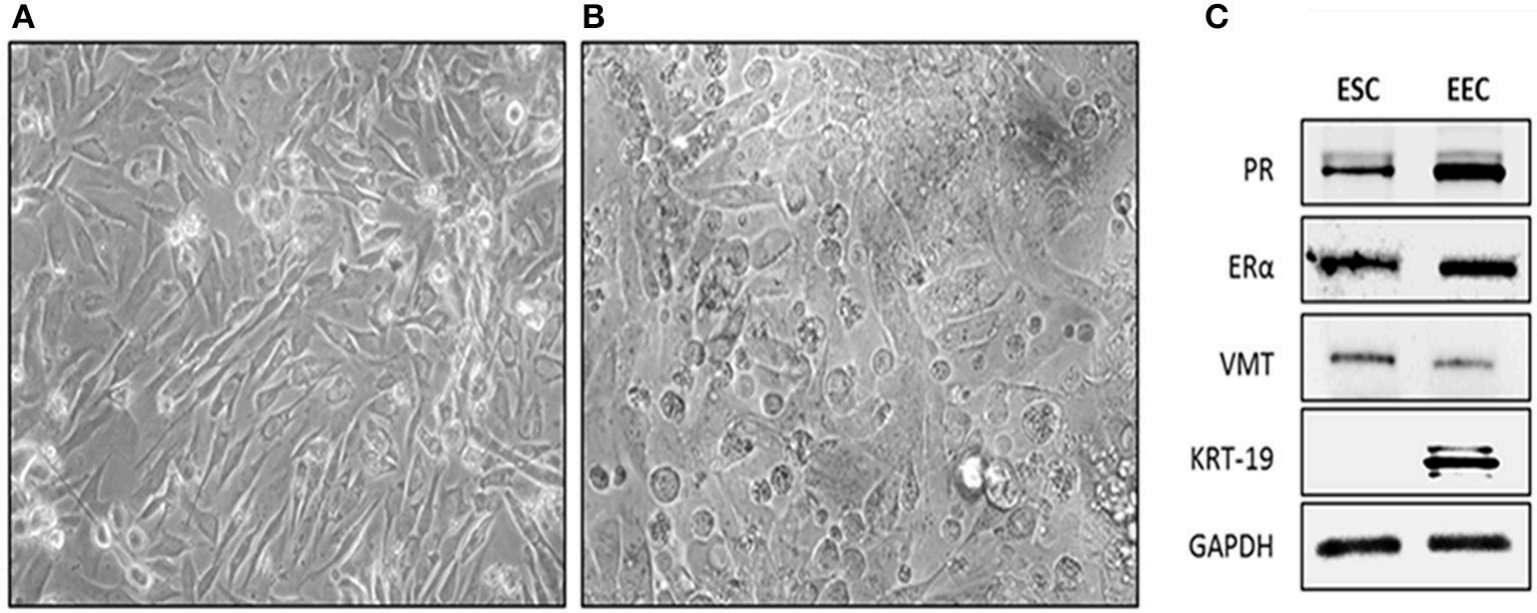
Isolation and characterization of human endometrial cells. Cells were obtained from endometrial biopsies and cultured in DMEM-F12 supplemented with 10% FBS. **(A)** Phase contrast microscopy of Endometrial Stromal Cells (ESC), cells appeared flattened and elongated with a fibroblast-like shape. Photo taken 4 day after plating (20X). **(B)** Phase contrast microscopy of EEC cells appeared cuboidal. Photo was taken 4 days after plating. **(C)** Representative western-blot blot of Vimentin (VMT), Cytokeratin 19 (KRT-19), Estrogen Receptor alpha (ERα), Progesterone Receptor (PR), and GAPDH. ESC were VMT/ERα/PR positive. EEC were KRT-19/ERα/PR positive. GAPDH was used as loading control.

As previously described, we managed to obtained ESC and EEC cells in different flasks. In contrast to EEC, ESC appeared flattened and elongated with a fibroblast-like shape under phase contrast microscopy (Figures 1A,B). To corroborate the purity of cell cultures, we performed western blot of stromal and epithelial whole cells lysate against cytokeratin 19 and Vimentin. As expected, stromal cells were Vimentin (+), Cytokeratin (–), and the epithelial cells were Vimentin low, Cytokeratin (+) (Figure 1C). In addition, we evaluate the expression of estrogen (ER) and PR with the purpose to be able to use ESC for the study. EEC and ESC express both hormone receptors (Figure 1C).

### Cell migration assays

We measure the collective cell migration with two methodologies scratch and wound healing assays. The First one, measure the migration distance from the scratch of one group of cells, which is associated with the lateral line model. The second one, measure the closure of the area of the wound or scratch and this one is related with the sheet migration model. These correlated methodologies are not equivalents and both have different strengths and weaknesses (19).

#### Scratch cell migration assay

Scratch Cell Migration Assay was performed with a plastic razor scrape as previously described Montt-Guevara et al. (20). Briefly, a razor blade was pressed trough the confluent ESC monolayer to mark the starting line and cells were swept away on one side of the line. Cells were washed and further medium containing CS-FBS and cytosine β-D-arabinofuranoside hydrochloride (Ara-C, Sigma-Aldrich, USA, 10 μM) were added to prevent cell proliferation. Ara-C is a selective inhibitor of DNA synthesis that does not inhibit RNA synthesis. After 1 h the active treatments were added (T0). Fresh medium containing the active treatments were replaced every 24 h and the cultivation was continued for 48 h (T48). Cells were photographed in five random fields per condition with Olympus BX41 microscope, DP70 Olympus digital camera. Cell migration distance covered by the cells between T0 and T48 was measured using NIH ImageJ 1.51q software (NIH, USA) in three different areas of each photograph. Cell proliferation in presence of Ara-C was checked in preliminary experiments by MTS (3-[4,5-dimethylthiazol-2-yl]-5-[3-carboxymethoxyphenyl]-2-[4-sulfophenyl]-2H-tetrazolium, Abcam, England) (Supplementary Figure 1). The Proliferation Index (PI) was calculated as follows: [ESC treated (O.D.) at time X/ESC treated (O.D.) at time 0].

#### Wound healing assay

Wound Healing Assay was tested with culture-insert two well (Ibidi, Germany). Briefly, 20,000 endometrial cells were seeded with DMEM-F12 containing CS-FBS in both wells. After 48 h, ESC were washed two times with PBS and incubated with Ara-C. One hour later all culture-inserts were removed, creating a cell-free gap of ~500 μm, and culture medium with the active treatments were added (T0). Migration was monitored for 24 h (T24). To determine wound closure area, images were taken by phase contrast microscopy (Olympus) at time 0 and 24 h. The gap area at T0 and T24 were measured by NIH ImageJ software performing the following operations: a background subtraction operation and then using the hand detection command we outlined the leading cell path and finally measure area. Coverage areas were calculated as [(Area T0 – Area T24)/Area T0] and expressed relative to control cell at T24.

Scratch and wound healing assays measure the same phenomena: collective cell migration. However, the first one, measure the migration distance from the scratch of one group of cells, and that is related with the Lateral line model. The other, measure the closure of the area of the wound or scratch and that is related with sheet migration model. These methodologies are not equivalents but correlated and both have different strengths and weaknesses.

### Immunoblottings

After treatments, cells were lysed in buffer Tris-HCl 50 mM, pH 7.4, 1 mM EDTA, 1% IGEPAL, proteases inhibitor cocktail PIC (Sigma-Aldrich, USA), and phosphatase inhibitor cocktail PHIC-3 (Sigma-Aldrich, USA). Protein concentration was quantified using BCA method (Thermo Fisher Scientific, USA). Cells lysates were resolved by SDS-PAGE as previously described (21). Antibodies against p^T558^-Moesin (sc-12895), p^Y397^-FAK (sc-81493), PR (sc-539), Vimentin (sc-66002), ER-alpha (sc-8005), and GAPDH (sc-59540), as loading control were all purchased from Santa Cruz Biotechnology, USA; Cytokeratin 19 (KRT-19, MA5-15884, Thermo Fisher Scientific, USA). Primary and secondary antibodies were incubated with standard technique. Immunodetection was visualized by chemiluminescence and digitalized with Quantity One software (BioRad, USA). Optical densitometry (OD) analysis of the bands was performed using the NIH ImageJ software. OD values were expressed as the ratio of each band vs. their respective loading control.

### Immunofluorescence

ESC were grown on coverslips and exposed to different treatments. ESC were fixed in paraformaldehyde 4%, for 20 min and then incubated with quenching solution (50 mM NH_4_Cl in PBS) for 20 min. Permeabilization and blocking were performed with 3% bovine serum albumin in PBS/ 0.1% Triton X-100 for 30 min. Cells were incubated with primary antibodies against p^Tyr118^Paxillin (sc-365020, Santa Cruz Biotechnology, USA) and Vinculin (CP74, Calbiochem, USA) over-night at 4°C and Alexa 488 anti-mouse (A11029, Invitrogen, USA) was used as secondary antibody. In addition, cells were incubated 45 min with Texas Red-X Phalloidin (T7471, Invitrogen, USA) for F-actin. Nuclei were stained with 4′-6-diamidino-2-phenylindole (DAPI, D9542, Sigma-Aldrich, USA) and mounted with Ibidi mounting medium (Ibidi, Germany). Immunofluorescence was visualized using an Olympus BX41 microscope and recorded with a DP70 Olympus digital camera.

The quantitative analysis of the remodeling and thickness of actin fibers was performed on 20 cells per experimental condition, by using the NIH ImageJ software in two different ways. The first one involved two separate measures in each cell of 10 μm distance encompassing extracellular space, full thickness of the membrane and intracellular. The program provides a graph and the mean fluorescence intensity as pixel gray value of the areas identified as membrane or the cytoplasm. The membrane/cytoplasm ratio (M/C) was calculated. For the second one, we performed a background subtraction operation, and then using the hand detection command we selected the outer surface of the cell membrane and measured the whole cell RawIntDen (is the sum of the values of the pixels in the selection). Subsequently, we selected the inner surface of the cell membrane and measured the cytoplasm RawIntDen. After that, we calculated the M/C ratio as [(whole cell RawIntDen – cytoplasm RawIntDen)/cytoplasm RawIntDen].

### Statistical analysis

Each biological experiment was carried out at least three times on independent replicates with comparable results. Data were analyzed using GraphPad Prism 7 software (GraphPad Prims, USA) employing one-way ANOVA followed by Tukey's multiple comparisons test. Results are expressed as mean ± SEM. Differences at *p* < 0.05 were considered significant.

## Results

### UPA does not trigger moesin and FAK phosphorylation in ESC, but modulates the effects of E2 and P4

To evaluate how UPA may affect cytoplasmic alterations in ESC, we checked if UPA might interfere with activation/phosphorylation of Moesin (T558) and FAK (Y397), two major proteins that are responsible for actin re-shaping.

**Figure 2:**
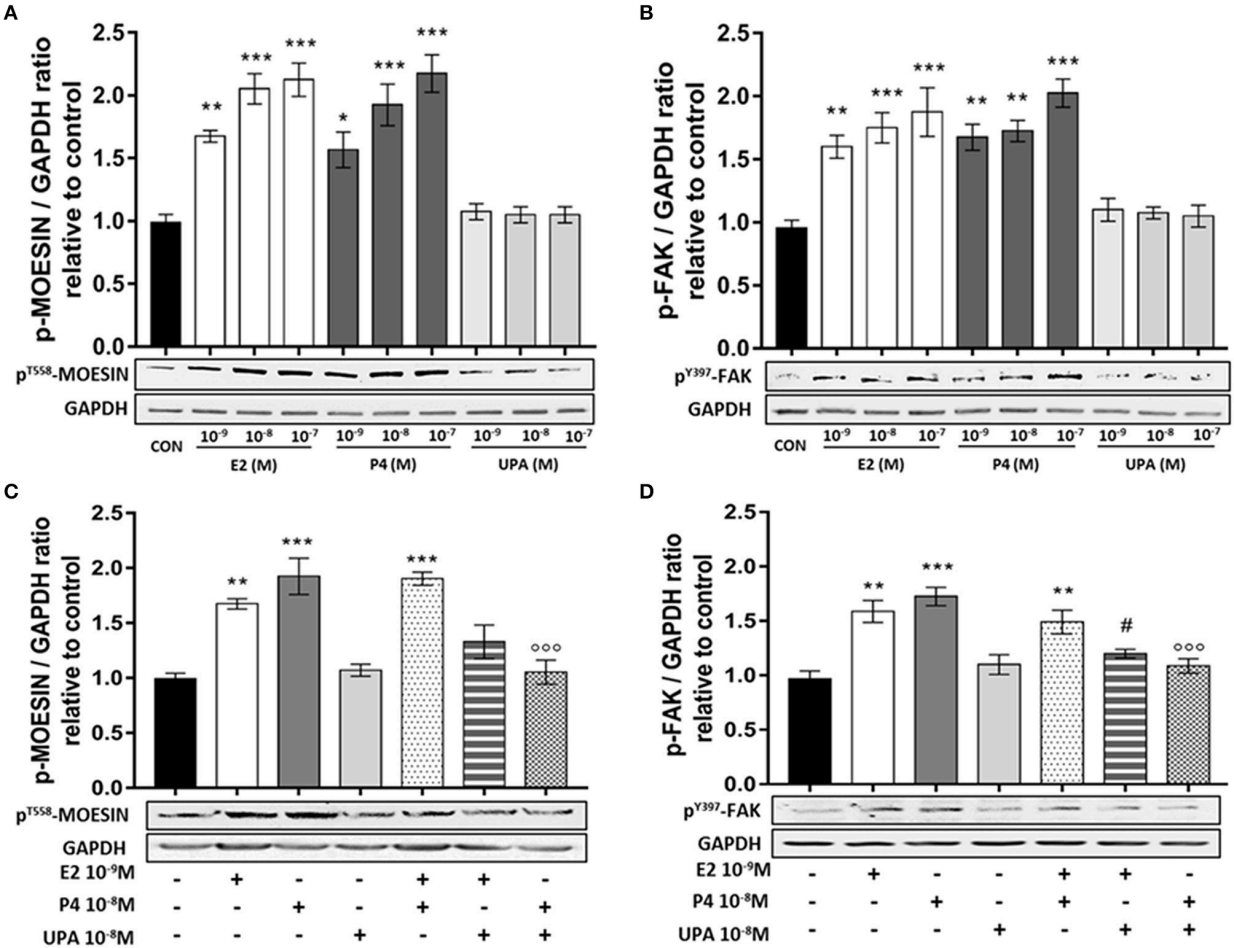
UPA inhibits Moesin and FAK activation induced by E2 and P4. ESC treated for 20 min with increasing concentration of E2, P4, UPA **(A,B)** and the combinations: E2+P4, E2+UPA, and P4+UPA **(C,D)**. Lower panels, show representative blot of p^T558^Moesin, p^Y397^FAK, and GAPDH. Upper panels, show the quantitative analysis of OD of western blot represented as mean ± SEM of p^T558^Moesin/GAPDH and p^Y397^FAK/GAPDH ratio relative to control. Four independent experiments were performed. The data were analyzed statistically by one-way ANOVA, followed by Tukey's multiple comparison test (^*^p < 0.05, ^**^p < 0.01, ^***^p < 0.001 vs. control); (^#^*p* < 0.05 vs. E2 10^−9^ M); (^°°°^*p* < 0.001 vs. P4 10^−8^ M).

ESC were treated with increasing concentration of E2, P4 and UPA (10^−9^ to 10^−7^ M) for 20 min. As expected, E2 and P4 induced a significant increase of ^T558^Moesin and ^Y397^FAK phosphorylation. On the contrary, no significant difference was observed in cells treated with UPA (Figures 2A,B).

Based on the previous results, we selected the following concentrations for successive experiments: E2 10^−9^, P4 10^−8^, UPA 10^−8^ M. ESC treated with E2+P4 displayed a rapid ^T558^Moesin phosphorylation compared to control, but no synergistic action was seen. When ESC were treated with E2+UPA or P4+UPA, ^T558^Moesin phosphorylation did not differ from the control. In addition, ESC treated with P4+UPA revealed a significant decrease of ^T558^Moesin phosphorylation compared to P4 (Figure 2C).

When we analyzed the phosphorylation of ^Y397^FAK, we found that ESC treated with E2+P4 displayed a significant ^Y397^FAK phosphorylation compared to control and no synergistic action was seen. Similarly, when ESC were treated with E2+UPA or P4+UPA we observed no induction of ^Y397^FAK phosphorylation compared to control. Moreover, UPA significantly decreased ^Y397^FAK phosphorylation induced by P4 and E2 (Figure 2D).

### UPA does not activate actin cytoskeleton rearrangement in endometrial stromal cells, but modulates the effects of E2 and P4

In order to characterize UPA cytoskeletal changes on normal ESC, we first studied whether it could modified the arrangement of actin filaments. We performed immunofluorescence studies to refine the localization of actin filaments with Texas Red-X Phalloidin.

Cells were treated with E2 10^−9^, P4 10^−8^, UPA 10^−8^, ICI 10^−6^ M (Fulvestrant, an ER inhibitor), MIF 10^−5^ M (Mifepristone, a progesterone receptor inhibitor), and the combinations (E2+P4, E2+UPA, P4+UPA, E2+ICI, and P4+MIF) for 30 min to observe actin cytoskeleton rearrangement.

**Figure 3:**
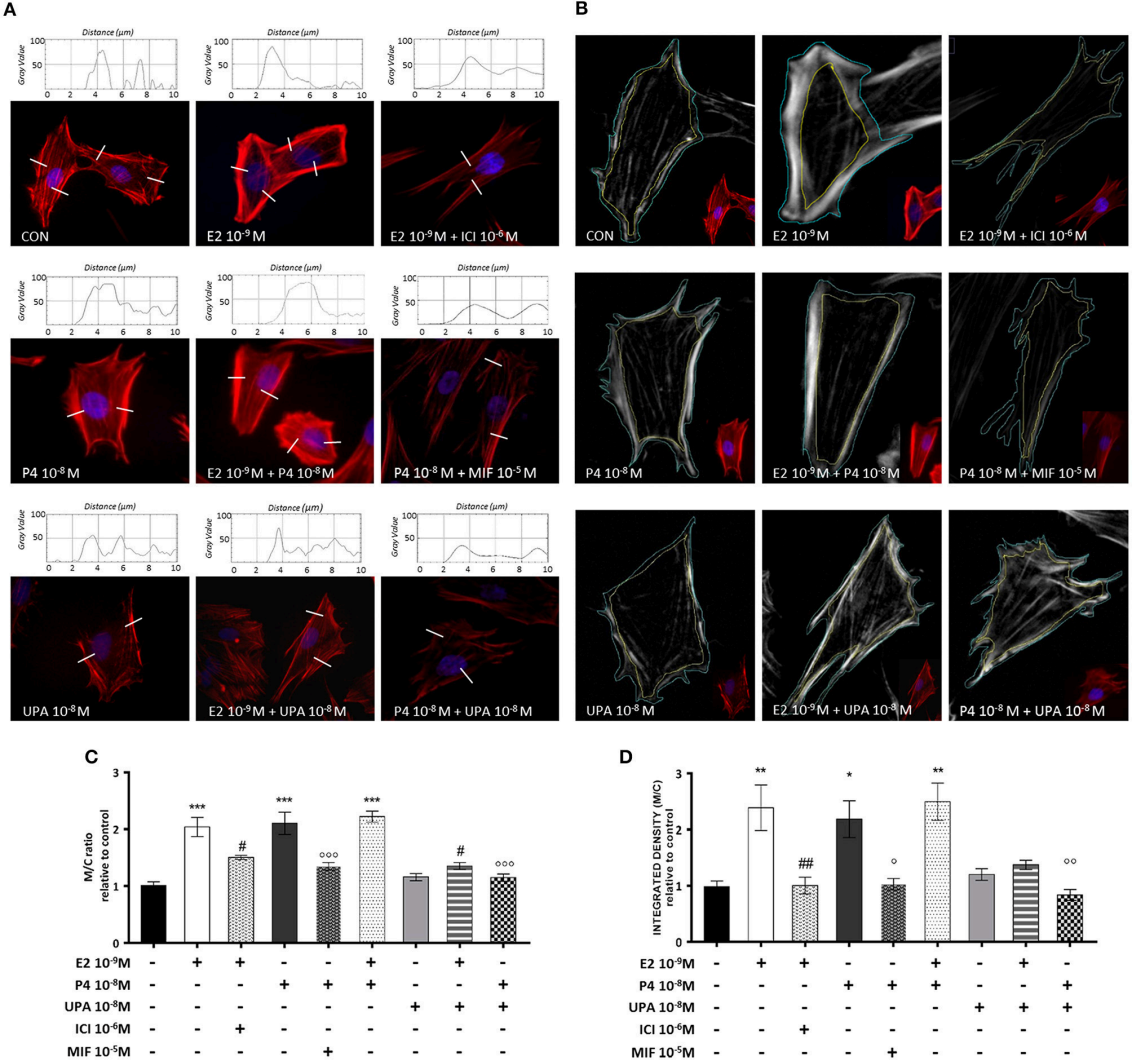
UPA inhibits actin rearrangement induced by E2 and P4. ESC treated for 30 min with E2, P4, UPA, ICI, MIF and the combinations. Actin fibers were stained with Texas Red-X phalloidin (red), and nuclei were counterstained with DAPI (blue). **(A)** Representative immunofluorescence images and descriptive plot-profile of fluorescence intensity measured as gray value. **(B)** Representative immunofluorescence images with the selected outer and inner surface of the cell membrane. **(C,D)** Quantitative analysis graph of pixel intensity represented as mean ± SEM of membrane/cytoplasm ratio (M/C ratio) relative to control. Three independent experiments were performed. The data were analyzed statistically by one-way ANOVA, followed by Tukey's multiple comparison test (^*^p < 0.05, ^**^p < 0.01, ^***^p < 0.001 vs. control); (^#^*p* < 0.05, ^##^*p* < 0.01 vs. E2 10^−9^ M); (°*p* < 0.05, ^°°^*p* < 0.01 ^°°°^*p* < 0.001 vs. P4 10^−8^ M).

Control cells displayed mainly longitudinally arranged actin fibers with regular cell borders. In addition, non-substantial changes were observed in cells treated with E2+ICI, P4+MIF, UPA, E2+UPA, or P4+UPA. However, when cells were treated with E2 and P4 or the combination (E2+P4), we observed actin filaments reorganization in the proximity of the cell membrane (Figures 3A,B).

We analyzed the membrane/cytoplasm (M/C) ratio by two different methods with comparable results. As expected, cells treated with E2, P4 and the combination, showed a significant increase on M/C ratio compared to control. The effects of E2 and P4 were counteracted by their specific inhibitors. UPA counteracted the actions of E2 and P4 on actin remodeling. The inhibition associate with UPA was analogous to that induced by ICI or MIF (Figures 3C,D).

### P4 and E2, but not UPA, trigger vinculin and p^y118^paxillin localization at the cell membrane

It is documented that focal adhesion complexes formation is associated to actin rearrangement, as an early step in cell migration (22–24). For this reason, we chose to study two proteins that participate in focal adhesion complexes, Vinculin and Paxillin. The first one is involved in the linkage of integrins, matrix adhesion molecules, to the actin cytoskeleton. The second one is a scaffolding protein activated by phosphorylation on Y118 residue; it serves as a platform for the recruitment of several regulatory proteins that modulate the assembly and disassembly of the focal adhesion complexes, linked to actin rearrangement. We investigated whether UPA could modulate focal adhesion complexes formation in the presence of E2 or P4.

ESC were treated with E2 10^−9^, P4 10^−8^, UPA 10^−8^, ICI 10^−6^, and MIF 10^−5^ M and their combinations for 48 h. Then cells were fixed and immunofluorescence was performed. Images were taken with a 100x objective magnification. All images were subjected to the same parameters values for background subtraction with the background correction plugin of imageJ software.

**Figure 4:**
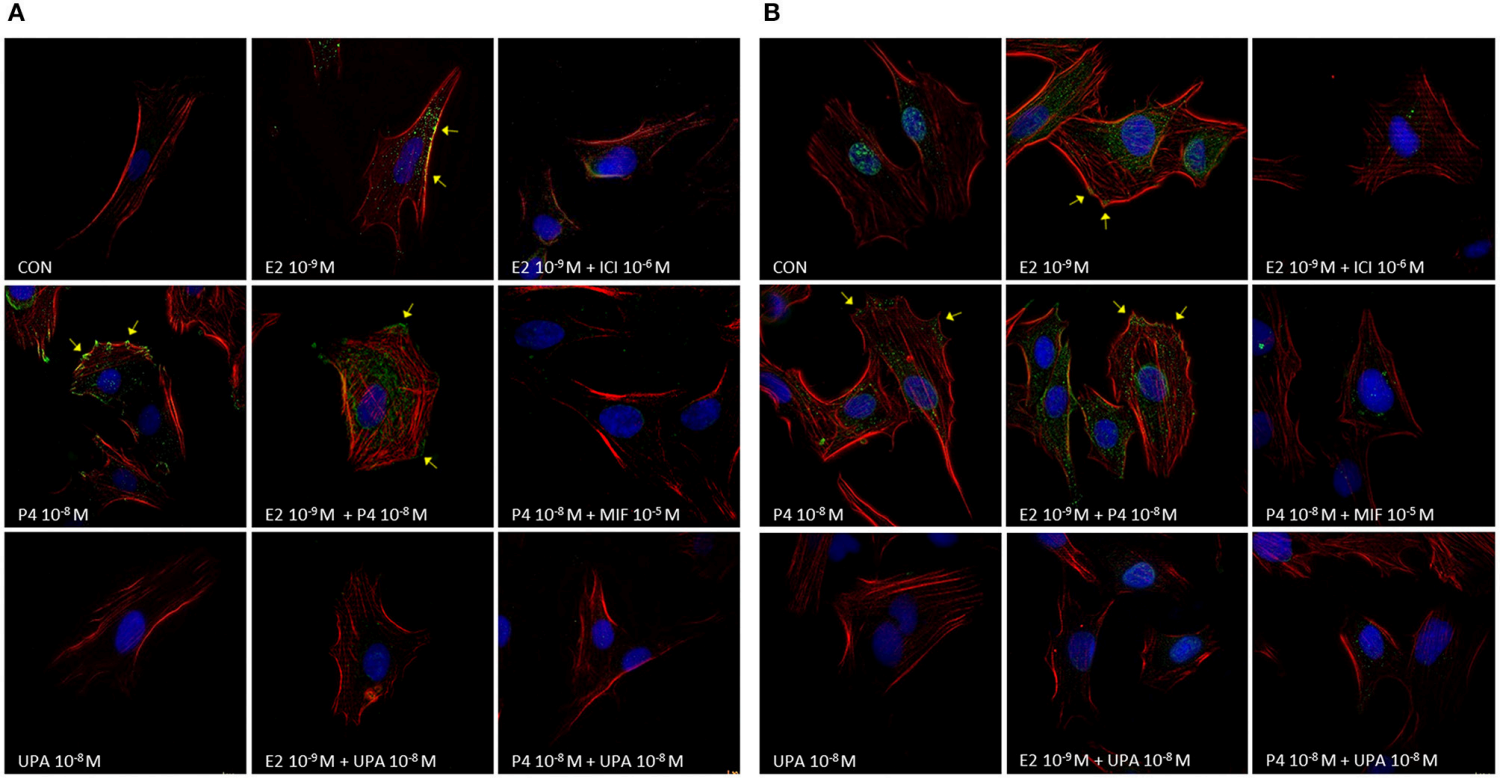
UPA inhibits the formation of focal adhesion complexes induced by E2 and P4. Representative merge immunofluorescence images from ESC treated for 48 h with UPA, P4, E2, ICI, MIF, and the combinations. Cells were fixed and incubated with **(A)** anti-Vinculin or **(B)** anti-p^Y118^Paxillin linked to Alexa Fluor 488 (green), F-Actin was stained with Texas Red-X phalloidin (red), and nuclei with DAPI (blue). Yellow arrows indicate Vinculin and p^Y118^Paxillin localization as discrete punctuated pattern, typical of focal adhesion points.

We observed that E2, P4, and E2+P4 treatments stimulated the localization of Vinculin and ^Y118^Paxillin phosphorylation as discrete punctuated pattern (typical of focal adhesion complexes) at the cell membrane (Figures 4A,B, yellow arrows). On the contrary, cells treated with UPA, ICI, MIF, and combinations like E2+UPA, P4+UPA, E2+ICI, and P4+MIF did not show the recruitment of Vinculin or ^Y118^Paxillin phosphorylation to the plasma membrane (Figures 4A,B).

### UPA does not stimulate ESC migration, but modulates the effects of E2 and P4

To assess if UPA cytoskeletal changes could affect cell motility of human ESC we performed cell migration assays.

**Figure 5:**
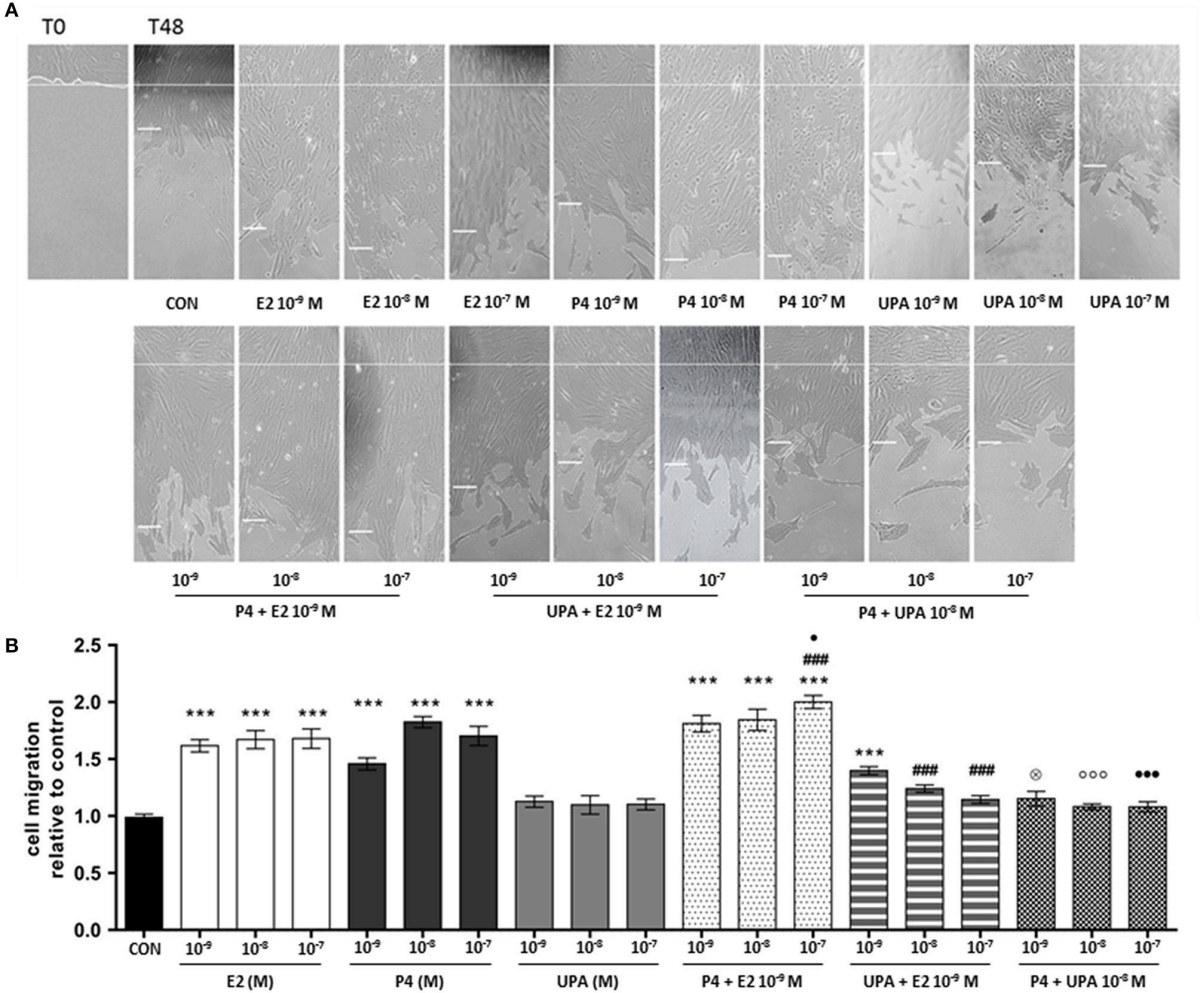
UPA inhibits ESC motility induced by E2 and P4. Cells scratch migration assays were performed with a razor blade pressed trough the confluent ESC monolayer to mark the starting line, then cells were swept away on one side of that line. ESC were treated with increasing concentration of UPA, P4, E2, and the combinations for 48 h. **(A)** Representative ESC migration images taken at 0 and 48 h. The short white lines represent the average distance of three measurements in the same image. **(B)** Quantitative analysis of cell migration represented as the mean ± SEM migration distance from the starting line relative to the control. Five independent experiments were performed. The data were analyzed statistically by one-way ANOVA, followed by Tukey's multiple comparison test (^***^p < 0.001 vs. control) (^###^*p* < 0.001 vs. E2 10^−9^ M) (^⊗^*p* < 0.05 vs. P4 10^−9^ M) (^°°°^*p* < 0.001 vs. P4 10^−8^ M) (^∙^*p* < 0.05, ^∙∙∙^*p* < 0.001 vs. P4 10^−7^ M).

ESC were treated with increasing concentration of E2, P4, and UPA (10^−9^ to 10^−7^ M) and combinations for 48 h. As expected, cells treated with P4 and E2 significantly increased cell migration from the lowest dose used. However, UPA treatment did not promote cell migration (Figure 5A).

For the first combination, E2+P4, we found that E2 (10^−9^ M) with increasing concentration of P4 (10^−9^ to 10^−7^ M) significantly stimulated cell migration compared to control. Moreover, cells treated with E2+P4 10^−7^ M had significantly higher migratory effect compared to E2 and P4 alone (Figure 5B).

For the second combination, E2+UPA, we found that E2 (10^−9^ M) with increasing concentration of UPA (10^−9^ to 10^−7^ M) only E2+UPA 10^−9^ M significantly increased cell migration compared to the control. However, UPA (10^−8^ to 10^−7^ M) counteracted in a concentration-dependent manner cell migration induced by E2. Finally, UPA significantly antagonized cell migration induced by P4 (Figure 5B).

To corroborate the results obtained in the previous experiments, we decided to evaluate cell migration with wound healing assay micro-inserts system. Images were taken at 0 and 24 h (Supplementary Figure 2A). We confirmed that E2, P4, and E2+P4 significantly stimulate wound closure. Once more, UPA did not show significant differences compared to control. We also observed a significant decrease on wound closure when we compared E2+ICI vs. E2 and P4+MIF vs. P4. Similarly, when cells were treated with E2+UPA and P4+UPA, a significant reduction of cell migration compared to E2 and P4 was found (Supplementary Figure 2B).

## Discussion

Human endometrium is the inner mucosa of the uterus, which undergoes cycles of regeneration (cell proliferation and migration), differentiation, and shedding during a woman's reproductive life. Moreover, the endometrium is one of the most responsive tissues to sex steroid hormones like progesterone (P4) and 17β-estradiol (E2) (25). Reciprocal paracrine signaling driven by fluctuating sex steroid hormones determines the stromal-epithelial cell functional expression patterns, proliferation state and rate of apoptosis (26). These dynamic changes on the endometrium are related to a set of cellular processes that happens during menstrual cycle, pregnancy, or the development of endometrial disorders (13). For these reasons, the isolation of ESC from healthy patients is an interesting *in vitro* model to study the effects of the SPRM, UPA.

The histopathological endometrial changes found in patients treated with UPA have been partially characterized (2, 9, 27, 28). These reversible endometrial modifications, have been described as benign, non-proliferative histologic features of the endometrium, and named Progesterone Receptor Modulator-Associated Endometrial Changes (PAECs) (10, 11). The cellular basis for such processes is unclear, but may well be based on altered cell cytoskeleton dynamics.

In this study, we present evidence that UPA modulates E2 and P4 effects on actin filaments rearrangement and on phosphorylation status of FAK and Moesin. In addition, we demonstrate that UPA does not promote the recruitment of vinculin and p^Y118^Paxillin to the leading edge of the plasma membrane as a discrete punctuated pattern, typical of focal adhesion points. These findings may suggest that UPA alters cell morphology by interfering with the cytoskeletal action of E2 and P4 in the endometrium.

In the recent past, the role of FAK, Paxillin, and Moesin in relationship with sex steroids in the biology of normal and cancer cells has been the subject of extensive investigation (29–32). Membrane-Organizing Extension Spike protein (Moesin) functions as a cross-linker between plasma membrane and the actin cytoskeleton. Focal Adhesion Kinase (FAK) is a non-receptor tyrosine kinase that regulates the formation of focal adhesion complexes, which provide anchoring sites for cell attachment to the extracellular matrix. Our group and others have reported diverse possible mechanistic explanations on how E2 and P4 regulate these biological processes (30, 31, 33–36). Estrogens receptors (ER) and PR recruit G-alpha-13 and promote the activation of the Src/FAK/Paxillin complex and its interaction with Vinculin, leading to N-WASP/ARP2/3 complex activation. On the other side, ER, and PR activate the G-alpha-13/Src/Rho-A/Rock-2/Moesin cascade. Both pathways promote cytoskeletal remodeling that leads to cell migration and regulate different cellular functions in endometrial cells (30, 37–39).

A recent observational study conducted by Whitaker et al., in women developing PAEC during treatment with UPA, shows that UPA alters the expression pattern of both PR and androgen receptors, decreasing endometrial cell proliferation rate (40). From *in vivo, in vitro*, and pre-clinical studies, we now know that UPA binds with high affinity to PR (41, 42), and in a weak manner to ER (43). This could be an explanation for the modulation of cell migration and actin filaments reorganization by UPA in presence of E2 and P4. Based on our results, PAECs could be related to the interference of UPA on cytoskeleton rearrangement modulated by E2 and P4 on ESC. Consequently, this would reinforce the idea that PAECs are not related to proliferative actions. However, why not all subjects develop PAECs upon expose to UPA is uncertain and the impact of this phenomenon on bleeding control is unknown.

Understanding the molecular basis of UPA actions on the endometrium may turn out to be relevant for several potential clinical applications, including endometriosis, adenomyosis, or estrogen/progesterone sensitive cancers. Studies suggest that UPA is a promising endocrine therapy for hormone-sensitive tumors like breast or endometrial cancer. Particularly, Esber et al., found anti-proliferative and pro-apoptotic effects in patient-derived breast cancer xenografts in nude mice exposed to UPA (44, 45). Other groups observed that UPA is able to reverse E2 and P4 actions by decreasing breast cell proliferation and hormone receptor expression on BRCA1-mutated breast tissue xenografted in mice; suggesting that a subset of women with BRCA1 mutations could be candidates for UPA treatment as a preventive breast cancer strategy (46). These studies are therefore the proof-of-concept that the selective antagonism with PR of UPA could be used in other clinical settings.

In conclusion, we demonstrate that UPA interferes with E2 and P4 actions in ESC by decreasing actin cytoskeleton rearrangement, focal adhesion formation, and reducing FAK and Moesin phosphorylation/activation. Moreover, this corresponds to the inhibition of cell motility. These findings add to our current understanding of the bio-molecular mechanism of actions of UPA and SPRMs on endometrial cells and highlight how cytoskeletal actions of UPA could be of interest to its clinical actions.

## Author contributions

JS, MMG, and TS conceived the study design, performed data interpretation, and manuscript preparation. MM, JS, GP, and MF performed the molecular studies. AG and ADG assisted in the manuscript preparation. All authors read and approved the final manuscript.

### Conflict of interest statement

The authors declare that the research was conducted in the absence of any commercial or financial relationships that could be construed as a potential conflict of interest.

## Supplementary material

Supplementary Figure 1 Effects of 10 μM Ara-C on ESC proliferation. ESC were treated with E2 10^−9^, P4 10^−8^, UPA 10^−8^, ICI 10^−6^ M. and MIF 10^−5^ M and the combinations for 72 h. Proliferation was measured by MTS assay every 24 h. Results are shown as the mean ± SEM of proliferation indexes (PI) of three experiments performed in triplicate. Data were analyzed statistically by one-way ANOVA. Cells incubated with Ara-C did not have significant effect on PI.

Supplementary Figure 2 UPA modulates the effects of E2 and P4 on ESC motility, similar to ICI and MIF. Wound Healing micro-inserts assay, was tested with culture–insert 2 well. ESC were treated with UPA, P4, E2, ICI, MIF, and the combinations for 24 h. **(A)** Representative phase contrast microscopy image taken at 0 and 24 h. **(B)** Quantitative analysis graph of cell migration represented as mean ± SEM of the percentage of wound closure area relative to the control. Three independent experiments were performed. The data were analyzed statistically by one-way ANOVA, followed by Tukey's multiple comparison test (^**^*p* < 0.01, ^***^*p* < 0.001 vs. control) (^##^*p* < 0.01 vs. E2 10^−9^ M) (°*p* < 0.05, ^°°^*p* < 0.01 vs. P4 10^−8^ M).
