# Supplementary figures and images for: Ulipristal Acetate Interferes With Actin Remodeling Induced by 17β-Estradiol and Progesterone in Human Endometrial Stromal Cells
Source: Front Endocrinol (Lausanne). 2018 Jun 27;9:350. doi: 10.3389/fendo.2018.00350 (PMC6036300; doi:10.3389/fendo.2018.00350)

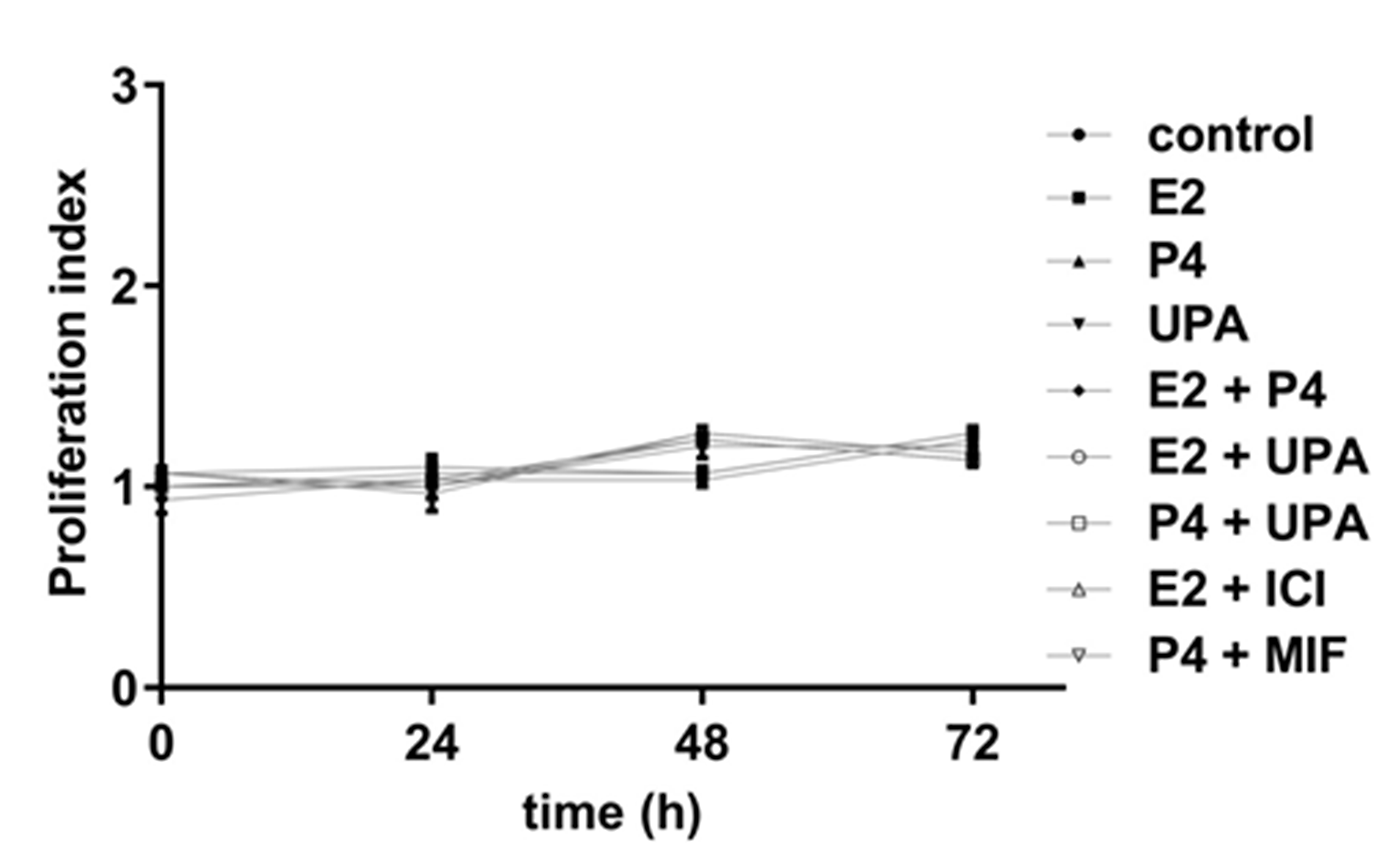

Supplement: Supplementary Figure 1 — Effects of 10 μM Ara-C on ESC proliferation. ESC were treated with E2 10−9, P4 10−8, UPA 10−8, ICI 10−6 M. and MIF 10−5 M and the combinations for 72 h. Proliferation was measured by MTS assay every 24 h. Results are shown as the mean ± SEM of proliferation indexes (PI) of three experiments performed in triplicate. Data were analyzed statistically by one-way ANOVA. Cells incubated with Ara-C did not have significant effect on PI. [file Image_1.TIF]

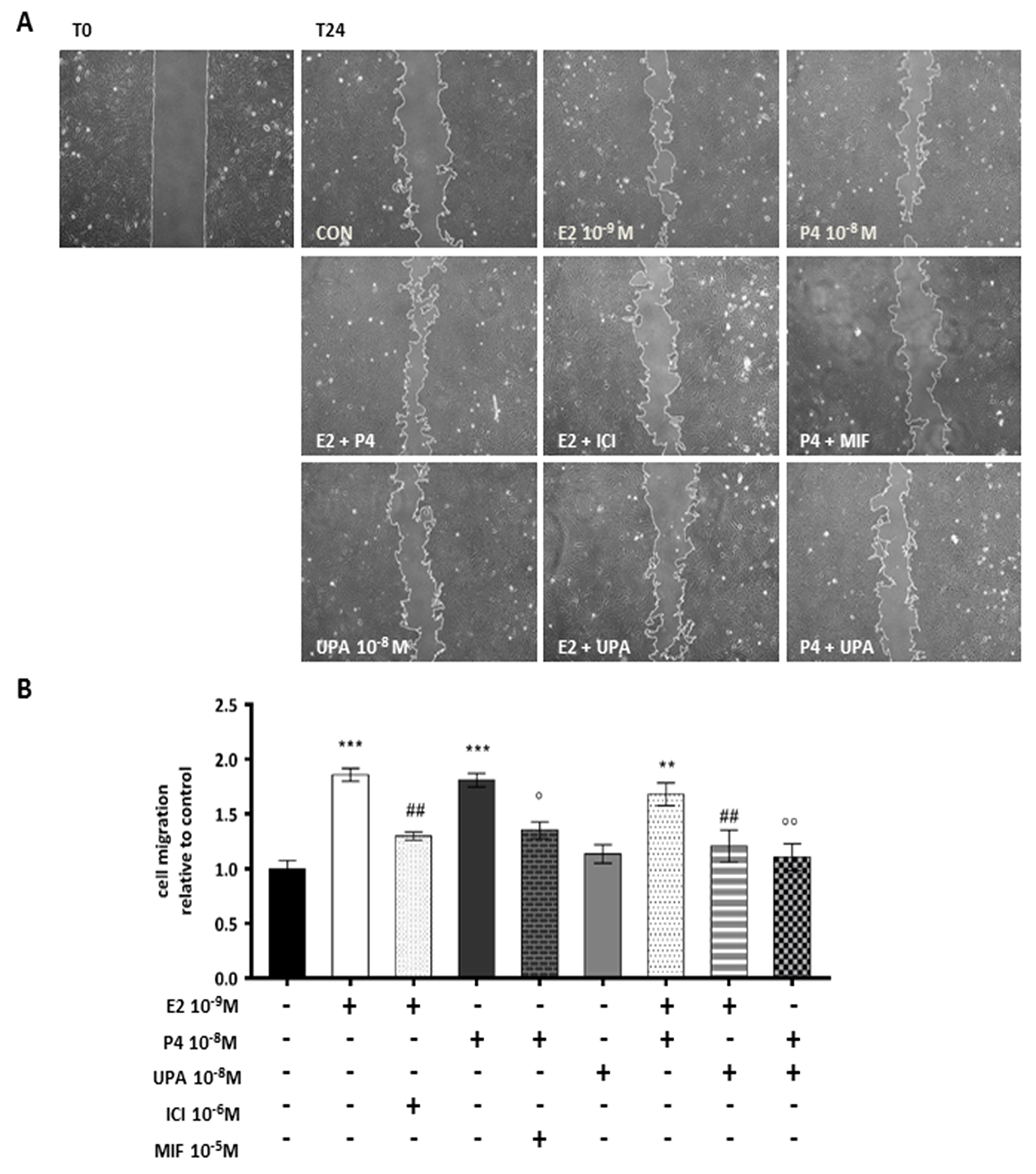

Supplement: Supplementary Figure 2 — UPA modulates the effects of E2 and P4 on ESC motility, similar to ICI and MIF. Wound Healing micro-inserts assay, was tested with culture–insert 2 well. ESC were treated with UPA, P4, E2, ICI, MIF, and the combinations for 24 h. (A) Representative phase contrast microscopy image taken at 0 and 24 h. (B) Quantitative analysis graph of cell migration represented as mean ± SEM of the percentage of wound closure area relative to the control. Three independent experiments were performed. The data were analyzed statistically by one-way ANOVA, followed by Tukey's multiple comparison test (**p < 0.01, ***p < 0.001 vs. control) (##p < 0.01 vs. E2 10−9 M) (°p < 0.05, °°p < 0.01 vs. P4 10−8 M). [file Image_2.TIF]
